# Older Adults’ Perceptions of Health, Quality of Life, and Access to Health Promotion Initiatives for Active Aging: A Qualitative Approach

**DOI:** 10.3390/ijerph22121796

**Published:** 2025-11-27

**Authors:** José Antonio Bicca Ribeiro, Breno Berny Vasconcelos, Kamila Bierhals Fernandes, Inácio Crochemore-Silva, Cristine Lima Alberton

**Affiliations:** 1 School of Physical Education and Physiotherapy, Federal University of Pelotas, Pelotas 96055-630, Brazil; jantonio.bicca@gmail.com (J.A.B.R.); brenobvasc@gmail.com (B.B.V.); kabierhals@gmail.com (K.B.F.); 2 Graduate Program in Physical Education and Graduate Program in Epidemiology, Federal University of Pelotas, Pelotas 96020-220, Brazil; inacioufpel@gmail.com; 3 Red Interuniversitaria de Envejecimiento Saludable, Latinoamérica y Caribe (RIES-LAC)

**Keywords:** older adults, physical activity, wellness, healthy ageing, social determinations of health, qualitative research

## Abstract

(1) Background: Health promotion initiatives based on physical activity (PA) play a crucial role in fostering active aging. However, engagement in such initiatives is related to individual subjectivities that vary according to different social backgrounds among older adults. This study aims to explore older adults’ perceptions of their health and quality of life after participation in a PA program, as well as the factors influencing their access to health promotion services and initiatives. (2) Methods: This is a qualitative study based on two focus groups conducted in June 2025 at the University involving 14 older adults (11 women; mean age: 68 years), participating in a supervised PA program in a city in southern Brazil. (3) Results: The themes identified from the Thematic Analysis were Health, aging, and quality of life; Engagement with PA; and Perspectives on health promotion strategies. A positive perception of health and quality of life was identified as being related to participation/engagement in a PA program. Access to health services is facilitated; however, waiting times for care are long, especially for more specific diseases. (4) Conclusions: Although participation in a structured PA program influences the positive perception of health and quality of life, socioeconomic and structural barriers limit equitable access to these services, revealing the need for integrated public policies to address health inequalities and support active aging.

## 1. Introduction

Population aging has accelerated significantly, making older adults the fastest-growing age group worldwide. According to the World Health Organization (WHO), in 2020, the number of people aged 60 years or older surpassed the population of children under the age of five [1]. In Brazil, the 2022 Demographic Census conducted by the Brazilian Institute of Geography and Statistics (IBGE) revealed an unprecedented aging trend, with more than 10% of the population aged 65 or older [2]. However, longevity does not necessarily translate into a fulfilling life, as multiple factors, whether social, cultural, or economic, can influence the aging process. Given this scenario, it is essential to understand the impacts of this phenomenon on life opportunities, quality of life, and life expectancy, since aging involves not only physiological changes but also social, emotional, and behavioral transformations [3].

Over time, individual aging has been associated with a gradual decline in physical and cognitive capacities, increasing the risk of disease [4]. Studies in the field of health and aging indicate that physical activity is an essential action for promoting health and enhancing the quality of life among older populations [5,6]. For example, the current global consensus guidelines from the International Conference on Frailty and Sarcopenia Research (ICFSR) recommend a comprehensive exercise prescription, including aerobic, resistance, balance, and flexibility training, to optimize healthy longevity in this population [7]. These practices have been shown to contribute both to the prevention and management of various diseases, in addition to their effectiveness as a non-pharmacological strategy for addressing a range of illnesses and comorbidities [8].

Although scientific evidence supports the relevance of physical exercise practice as a tool for promoting both individual and collective health, access to these practices remains unequal, influenced by factors such as gender, race, income, and geographic location [9]. The WHO highlights that women, older adults, low-income populations, individuals with chronic diseases, and marginalized groups, such as Indigenous peoples and residents of rural areas, have less access to physical activity [10]. In the Latin American context, socioeconomic and cultural inequalities exacerbate these barriers even further [11]. It is also worth noting that inequalities in access to health promotion strategies, such as those related to gender and age, affect a significant portion of the Latin American population, with women and older individuals exhibiting lower levels of total physical activity, leisure, and occupational activity, for example [9,12].

Brazil, one of the most unequal countries in the world, faces major and persistent challenges in this regard [9,13]. Access to active aging policies, especially physical activity programs, still seems distant, considering a country like Brazil, which may be due to low participation among Brazilian older adults or the marked social inequalities associated with them. The involvement of these individuals in these activities is linked to gender, education, and socioeconomic level, with men, those with higher education, and those with higher incomes tending to participate more in social, physical, and productive activities. When examining only the prevalence of physically active older people in their leisure time, a study conducted by Sousa et al. [5] revealed that this value remains low (13.1%), compared to the estimate for the global older population (45%). Furthermore, if we analyze all the social inequalities that permeate the indicators of active aging, the greater accumulation of educational and income is intimately linked to better health and well-being, since it enhances and qualifies access to activities, whether they are leisure, intellectual, and/or social activities [12,14].

However, the effects related to access to physical practices are not universal and may vary depending on the environmental conditions, social class, transitions experienced in old age, and quality of life, which is directly related to the interests, goals, expectations, and lifestyle of older adults [15]. This highlights the urgent need to generate knowledge across various sectors of society, particularly in public health, with the aim of supporting this population and improving both basic living conditions and healthy habits, including physical and mental well-being. Understanding different perceptions and experiences allows us to engage in reflection and deepen our comprehension of phenomena that occur in specific contexts but can be perceived on a broader, macro scale. In this sense, we employed a qualitative approach within critical health research as a key element of this study, aiming to gain a broader and more sensitive understanding of the multiple dimensions that influence health, particularly among older adults, while identifying essential elements for the development of more effective and humanized interventions [16,17]. Furthermore, as argued by Minayo [18], qualitative research is capable of addressing understanding, interpretation, and dialectics, elements that enable an examination of the subject’s subjectivities. It may also explore various methods for examining and reflecting on this reality [19].

Therefore, effective and equitable public policies are urgently needed, especially for the most vulnerable populations, to build more inclusive and sustainable cities, and promote the dignity of older adults, including housing, employment, social protection, and access to healthcare, as well as specific actions aimed at health promotion and social inclusion [20,21]. Considering the above, the ongoing demographic shift toward population aging and the well-established benefits of physical activity for older adults, this study aims to analyze older adults’ perceptions of their quality of life, health, and the factors that influence their access to health promotion initiatives based on participation in a physical activity program.

## 2. Materials and Methods

We used a qualitative approach [16,18,22], seeking to explore the topic in depth and examine its subjective aspects.

The study sample consisted of older adults (aged 60 years or older) who participated in supervised physical exercise programs linked to a randomized clinical trial registered at ClinicalTrials.gov (NCT06380413). The trial was a single-center, two-arm, parallel-group study involving an intervention and an active control group, with quantitative assessments of physical and mental health outcomes [23]. The study was conducted from August 2024 to May 2025 at the School of Physical Education and Physiotherapy of the Federal University of Pelotas (ESEF/UFPel), situated in the city of Pelotas, southern Brazil. In this trial, the intervention group participated in a 12-week program that combined Tai Chi Chuan practice with walking sessions, whereas the active control group performed only walking, both delivered in twice-weekly supervised sessions. Upon completing the intervention, all participants were invited to continue their practice in a free community-based exercise program centered on Tai Chi.

All individuals from the final recruitment wave of both groups (*n* = 20) were invited to participate in this qualitative component, and 14 participants accepted and attended the data collection session, which occurred in June 2025. The participants who agreed to take part were precisely those who continued attending the free university extension Tai Chi classes after completing the research intervention, while the remaining individuals declined due to program discontinuation or personal scheduling constraints.

All participants signed an informed consent form prior to participating in the study. The research was approved as an amendment to the original protocol, including this focus group analysis, by the Research Ethics Committee of the Federal University of Pelotas (CAAE 78972024.7.0000.5313).

Data were collected through a focus group approach, a qualitative technique designed to gather information from participants via group interaction. These sessions were conducted by an experienced moderator using a semi-structured interview guide (see Table 1), which aimed to capture participants’ perceptions [19]. Given the number of participants, we chose to organize two focus group sessions, each with seven participants, which facilitated participant engagement and improved session management. Each focus group was held in a private room at the university. All sessions were audio-recorded for subsequent transcription. Both focus groups were conducted on the same day and lasted approximately one hour each.

The groups were moderated by a researcher (J.A.B.R.), who had previous experience in this type of data collection, and was responsible for conducting the dialogue using the question guide presented in this text, which was specifically developed for this study. In addition, another researcher (K.B.F.), who had already been in contact with the group of older individuals during the randomized controlled trial, participated in the data collection as an external observer, noting relevant aspects of the responses as they emerged in the conversation and managing the recording equipment. The transcription was carried out by the group moderator, who was also responsible for the thematic analysis. Since he was present during data collection, he was able to grasp elements that aided in defining the themes presented, such as voice intonation, participant posture, and facial expressions.

Following transcription, the textual data (corpus) were imported into MAXQDA^®^ (Software for qualitative data analysis, VERBI Software version 24, Consult, Sozialforschung GmbH, Berlin, Germany) for organization. Data were analyzed using Thematic Analysis, a qualitative method widely used in health research that involves grouping responses based on recurring themes or categories related to the research questions [24].

The material was thoroughly read, and the initial step of the analysis involved identifying the most important and frequently recurring codes in participants’ statements (coding), which led to the development of initial analytical themes. Next, these elements were grouped by similarity to create secondary themes or subthemes. From there, the main themes that guide the analysis and are directly related to the study objective were developed. It is worth noting that data saturation was achieved through focus groups, as many similar responses were observed to each questionnaire question during those sessions. Additionally, responses were carefully allocated and reallocated until no new themes emerged and all topics were fully covered. Regarding data triangulation, it was used to support the analysis and understanding of subjects’ perceptions related to their social characteristics (sex, age, marital status, and occupation), thereby improving the understanding of the themes identified through thematic analysis.

## 3. Results

Table 2 shows the main characteristics of the participants, providing more context for understanding the findings. The participants were all older adults, with a mean age of 68 years (range: 62–76 years). Most participants were women (*n* = 11), with only three being men. Regarding marital status, four participants were single, four participants were widowed, three were married, and the other three were divorced. Most were retired (*n* = 12), although two women continued working as a hairdresser or a domestic worker to supplement their income. The remaining two participants received pensions or worked in paid jobs. During the focus group discussions, most participants reported living with family members, including spouses, children, or grandchildren, while only two participants lived alone. It is also worth noting that all participants were residents of the city of Pelotas, although they came from different neighborhoods, including both central and peripheral areas.

The themes that emerged from the thematic analysis were health, aging, and quality of life; engagement with physical activity; and perspectives on health promotion strategies (Table 3). In addition, their respective subthemes are represented and described in this section, along with excerpts from participants’ statements selected to exemplify each theme and subtheme.

### 3.1. Health, Aging, and Quality of Life

Participants were asked about their perceptions of health and quality of life. Their responses were grouped and categorized into two subthemes. The first relates to the presence of illnesses and comorbidities, which shaped how participants understood and evaluated their quality of life and health status. The second subtheme addresses the perspectives of older adults on the aging process itself, often intertwined with their perceptions of health and quality of life.

#### 3.1.1. Presence of Illnesses and Comorbidities

Participants reported a range of chronic health conditions, with hypertension being the most prevalent (*n* = 10). In addition, two participants reported having heart disease (e.g., arterial occlusion or previous myocardial infarction), three have diabetes, and four mentioned musculoskeletal conditions such as arthritis, arthrosis, or bursitis. One participant had undergone surgery to remove a prostate tumor, another reported having cataracts, and four mentioned receiving treatment for anxiety and depression.

Despite these illnesses and comorbidities, participants maintain a positive outlook regarding their health, as they are able to carry out their daily activities and engage in social interactions across various settings in everyday life. They also noted the attentive role of their families in supporting their health and medical care. It is worth noting that all participants reported being under medical care and following treatment for their illnesses. However, their concerns seemed more focused on managing existing conditions rather than on preventive health measures.

The following statements reflect the participants’ perceptions related to this subtheme.

“I’m a cardiac patient, right? I have heart problems. So, yeah... According to my son, who is a physical education teacher, I really needed to do some activities. And now that he’s a physiotherapist, he told me I needed to find something.” (ID2)

“I have high blood pressure, but it’s under control. My blood sugar is under control too. Until then, the machine wears out, but I’m doing very well.” (ID1)

“I have cataracts, need surgery, and I don’t see very well, you know? That’s the only difficulty I have, but let’s keep going.” (ID5)

#### 3.1.2. Perspectives on the Aging Process, Quality of Life, and Health

Although participants reported living with various chronic conditions and taking medications regularly, they expressed a positive perception of their health. The most commonly mentioned aspect contributing to this perception was autonomy in performing daily and routine activities. Other elements cited included healthy eating habits, a positive body image, sufficient energy to complete everyday tasks, and an overall sense of well-being, among others.

To the vast majority, reaching old age does not mean being excluded from decisions about their own lives, nor does it mean giving up on actions or activities because of potential challenges. They understand that aging is viewed as a life phase and identify possible difficulties that can be faced and overcome in pursuit of their goals. Most participants live with family members, which they consider an important factor in maintaining a good quality of life, such as being able to spend time with children and grandchildren or continuing to engage in social activities.

Another recurring theme cited by most older participants was their self-perception and the meaning they attributed to health and quality of life. Feeling good about themselves was consistently identified as a key element of positive health perception.

The following statements present the participants’ perspectives on this subtheme.

“Health, in terms of practical life, of daily life... It’s fundamental, right? So, we can live well, function well, and enjoy the time we have. Each stage, right? Knowing how to live life without health makes it harder. Not impossible, but harder. That’s when we start climbing the calvary.” (ID8)

“Not having to rely on others. If you can’t do those things, regardless of your son, your grandma, your mother, your granddaughter, or whoever you have now.” (ID6)

“Then, we are going to clear the way for everything. You go where you want, on your own. Not needing others. Not that you won’t need them, but being able to do things by yourself.” (ID10)

“We don’t depend on our sons or daughters, and we don’t depend on anyone else. We have our possessions. We go out, come back, and do everything on our own.” (ID11)

“And I believe that’s what it means to be healthy. As we age, people, your son or daughter looks at you and says, ‘You’re getting old, huh?’ But after this, today, my son or daughter looks at me and says, ‘Mom, you’re doing great.’” (ID6)

“So now, for me, it’s about gaining a little independence. I was comfortable before, but I didn’t do much, just watched life go by. What pressure! It’s the will to live, right? Health is the will to live, to do things, and there’s nothing stopping you, right? I think I don’t feel like I’m 69.” (ID9)

“To me, health is the balance between body and mind. I didn’t know much about my body before. That’s very important. And then everything else is a consequence. The will to live, to try new things, to explore, to share.” (ID14)

### 3.2. Engagement with Physical Activity

One of the topics addressed in the focus groups concerned older adults’ engagement with physical activity or exercise as a strategy for health promotion and active aging. The responses obtained in this theme were grouped and categorized into three subthemes, focusing on the importance and benefits attributed to the activities they engage in, the motivational factors and reasons for choosing specific practices, and the participants’ understanding of the main barriers or obstacles to participating in physical activity and exercise programs.

#### 3.2.1. Understanding the Importance and Benefits of Physical Activity

All participants were currently engaged in a physical activity program (Tai Chi and/or walking), as previously described in the study methods section. Of the 14 participants, only two reported participating in strength training in addition to the program, either on their own initiative or at the recommendation of family members or health professionals. Additionally, two participants mentioned using bicycles for active transportation in their daily routines, while eight reported walking occasionally during their leisure time.

The data suggest that participants’ understanding of the benefits of physical activity is primarily related to managing existing health conditions and positively affecting the aging process. They acknowledged the importance of having professional guidance from physical education specialists and expressed that regular physical activity contributes to improved health, quality of life, and overall well-being.

The following statements present the participants’ voices related to this subtheme.

“Because I believe physical activity doesn’t have an age. Because we only get older, but we don’t stop having desires and the will to achieve our dreams and things we couldn’t do before, because we had to work, we had responsibilities to meet.” (ID8)

“When I exercise, I feel fine. If I stop […], I start feeling my back again. Another interesting thing is that I used to have pain. I underwent treatment for many years with… an orthopedist, a physiotherapist […]. And I think that, through the activities we do, the exercises, one day, suddenly, I realized I wasn’t feeling pain anymore […] I can’t find it anymore; I haven’t felt the pain since. So, for me, that was wonderful.” (ID2)

“It’s improved because we get distracted, then realize we need to support ourselves to keep our balance. That’s the important part we’re learning now.” (ID3)

#### 3.2.2. Motivation for Practice

The reasons that led older participants to engage in physical activity were closely tied to their perception of the health benefits, as well as intrinsic personal motivations that support their continued participation. In addition to family encouragement, we identified a sense of belonging to a social group, which emerged as a key factor mentioned by most participants. Increased autonomy in performing daily tasks, improvements in physical capacities such as strength and balance, opportunities for interaction with instructors and monitors during activities, and a reduction in anxiety and stress symptoms were also cited as motivators for engaging in physical activity. Four participants specifically highlighted the support of their spouse or partner, as they attended the activities together and mutually encouraged each other. Two others mentioned the activity itself, particularly Tai Chi, as a source of motivation, due to its body–mind integration, in addition to promoting self-awareness.

Although this theme had focused on physical activity, seven participants also reported involvement in other social engagement groups (e.g., choir, sewing, or memory stimulation workshops). The motivations for participating in these activities were quite similar to those related to physical activity, suggesting a connection between routine choices and the pursuit of social and health-related well-being.

Additional statements underscore the importance of personal meaning, emotional connection, and social interaction as key motivators for sustained participation in physical activity.

“It’s something, […] that really aligns with my desire to improve myself, and how I connect with others. With the severity of my mother’s illness, I’ve been thinking about that a lot. If I have this opportunity that she never had, then why not? Maybe, indirectly, I can even help in some way.” (ID9)

“Now we get to meet new people and form new friendships to talk to. That’s something older people really miss—having the freedom to have conversations and make new friends. It makes a big difference. That’s it.” (ID11)

I thought I was doing well on my own. But then you join a group like this and see people doing even the smallest things that you can’t manage. That makes you think, ‘No, I’m going to try to do it.’ That motivates you. It pushes you to learn more. And there’s nothing better than this here.” (ID13)

“So, I think that is what happens with everything we come here; both the exercise and the time we spend with other people help us let go of some of that stress we carry. And I think it’s made a big difference in my life.” (ID12)

“One of the things that helped us get results so quickly was the instructor’s didactics. I had watched some Tai Chi videos and even told him: ‘The guys in those videos don’t come close to you’. Because teaching Tai Chi isn’t simple, but with him, we gradually understand it. And then he gets us involved, keeps talking to us. He’s very, very didactic. I think that’s a big reason we progressed so much in such a short time. There was real empathy.” (ID1)

#### 3.2.3. Perceived Barriers

The barriers reported by participants can be divided into personal and socioeconomic factors. Among the personal barriers, physical limitations in each participant stood out, particularly those related to health conditions that prevent certain corporal practices. For example, three older adults reported osteomuscular injuries (such as bursitis and tendinitis), and one mentioned cataract, which was an impediment for them for an extended period. Additionally, three participants highlighted caregiving responsibilities for family members (e.g., mothers or grandchildren), whose demands maintain a very busy routine, leaving little time for engaging in physical activity. Moreover, the COVID-19 pandemic was also mentioned as a disruptive factor, as some participants, despite being physically active, had to interrupt their activities during this period.

Regarding socioeconomic barriers, most participants (*n* = 10) reported being unable to cover the costs associated with structured physical exercise programs, such as those offered at a gym or accompanied by personal trainers. Since they are retired, their income is committed to other essential expenses in their hierarchy, making physical activity programs financially unfeasible. Two women also reported that, although retired, they continued to work to supplement their income and meet basic daily needs, further reinforcing their limited access to paid exercise programs.

These barriers are expressed in the participants’ own words in the following statements.

“I was already somewhat familiar with Tai Chi. I took a few trial classes and then practiced for about two months. It’s something I really enjoy. And for me, it came at a crucial time because I was very nervous and anxious. I left everything at my house and moved in with her [a family member], under unstable conditions, without even having a room of my own. I’ve been sleeping on the sofa bed in the living room. So, this is a way for me to clear my mind and not focus solely on that, because I also need to take care of myself. She has a caregiver, but I’m with her 24 h a day.” (ID6)

“Because I had been doing physical activities for a long time, but due to the pandemic, I stopped going to the gym. I stopped walking and everything else. I used to live on Duque de Caxias Street, right across from the barracks, and I used to walk there. I did everything on foot.” (ID4)

“But to pay for it, who can do it, being retired? I don’t know. Everyone has their own situation, right? But for us to pay for it, both of us, it would impact our budget. We would have to give something else up.” (ID2)

### 3.3. Perspectives on Health Promotion Strategies

Seeking a deeper understanding of the older adults’ perceptions regarding health promotion and social participation, participants provided a range of responses based on their diverse experiences. The data obtained from the focus group were grouped and categorized into two subthemes, one related to the aspects related to the participation of older adults in a supervised physical activity program and social settings aimed at promoting health and/or strategies to enhance the visibility and well-being of older individuals; and the other concerning to the access to and evaluation of the healthcare system, reflecting how well their needs are being addressed.

#### 3.3.1. Participation in Social Programs and Projects

In addition to the engagement in Tai Chi and guided walking sessions, three older adults mentioned having previously participated in other similar initiatives at the municipal level, whose main focus was to ensure equitable access to health promotion strategies. Two women also reported participating not only in a physical activity program, but also in other structured group activities (such as choir, sewing, and dance), which are systematically organized and supervised by the municipal government and other local universities (i.e., Catholic University of Pelotas), also offered free of charge to older adults.

It is also worth noting that most participants emphasized the value of participating in such programs, appreciating the opportunity to engage in various forms of physical activity. They mentioned various benefits resulting from their participation, including building relationships with other older adults, interacting with instructors and monitors, and improvements in their overall health, among others, as well as the positive impact on their daily life. They also expressed the hope that such initiatives would continue and that more opportunities would become available to reach a larger number of individuals in their age group.

Another aspect concerns the awareness of social program activities, with most participants reporting difficulty in locating information about such initiatives and the opportunities available. In many cases, they learn about them through conversations with friends or family members, who either inform or encourage them to join a specific activity.

The following statements present the participants’ perceptions related to this subtheme.

“Because it’s a way of showing concern for health, for each person’s being. We don’t need to be suffering from something to seek out a care service. We can focus on prevention instead of trying to manage problems later. According to my colleague’s statement, we also have poor health services and an inadequate education, right? But there are still some things worth saving, right? And there are still people who don’t have access to the resources that are available, right?” (ID9)

“A long time ago, when ESEF was next to the CEEE […]. I spent quite a bit of time there, almost the entire period. I was coming from work and heading straight there to fly. And that was the only project.” (ID3)

“I also take part in the occupational therapy project. Ah, it’s nice. It’s called ‘neurobics’, exercises for the brain. I do that too, at the Anglo building, with UNAPI. And I also take part in a medicinal plants group.” (ID4)

“The group is great too, right? When we arrive here and spend time together, and everyone’s always smiling, it’s a moment we relax, right? Pure relaxation with concentration. Because afterward we joke around, and he [the instructor] jokes with us too, but there comes a time for concentration.” (ID2)

“There are certain things that make us realize the future is now, right? So, we need to enjoy our future. Our future is today. We shouldn’t leave things for later. If we’re being given this opportunity by the university, I think we should seize it with both hands and hope there are other projects we can also participate in. It seems like when you join one, you can’t join another.” (ID11)

“I think it’s a social responsibility to take care of those in need, especially those who really need it, right? And offering quality of life, right? Quality of life in old age, which is what the third age is about. We have to enjoy most of it, right? Know how to enjoy the good things that come with aging, right? But with quality of life, right. There comes a moment when we don’t even know how old we are anymore.” (ID8)

#### 3.3.2. Use of the Healthcare System

The use of health services was also a topic mentioned by the participants in relation to managing their illnesses, which they viewed as a means to maintain their quality of life and overall well-being. Only three older adults reported having private health insurance, while the others rely on the Unified Health System (Sistema Único de Saúde—SUS), primarily through local primary healthcare services for routine follow-ups.

It is worth noting that, although many participants evaluated the service positively, others reported that, depending on the situation, the waiting time to see a specialist or undergo diagnostic tests is excessively long. A lack of information regarding how to use the SUS was also identified as an important barrier, along with the perception of inadequate care and insufficient public investment in health services for the older population.

The following statements reflect the participants’ perceptions related to this subtheme.

“Sometimes we feel […], sometimes we have a union, we pay for it, and there we get a discount for appointments. So, we go there and pay just a few reais for a consultation. But we avoid going even then, just not to spend money. And the exams are very expensive. I recently had one that cost 261 reais.” (ID8)

“Too much bureaucracy. That’s how I see it; I’m not sure if everyone feels the same way. I see politicians misusing public funds; that’s all we hear about. And nothing goes to healthcare, nothing to education. I blame the politicians, and the politics in Brazil.” (ID1)

“I went to the health clinic for a consultation. I was practically kicked out of there. I went on the wrong day. I didn’t know how it worked, what the process was. The guy just came at me aggressively, saying appointments are scheduled on Fridays. I had gone on a Tuesday. I told him, ‘I didn’t know, I had never been here, it’s my first time here. I’ll come back on Friday, what time should I come?’ And it’s a fight to even get… to even get information, right? Even a piece of information.” (ID2)

“Today, I rely on the SUS. For us, the SUS is a complex issue. I have been waiting for two years for surgery. Cataract surgery, right? I’m losing my vision. What can I do? It’s complicated for those who are salaried. I used to be able to pay for private care, but not anymore; it’s too expensive. But we go on, right? We wait in line.” (ID4)

“I have private health insurance. So, at the beginning of the year, I usually get a check-up.” (ID8)

## 4. Discussion

This study aimed to analyze older adults’ perceptions of their quality of life, their health, and the factors influencing their access to healthcare services and health promotion initiatives, with some insights into the aging process. Empirical data were collected through focus groups, as the qualitative approach allowed us to capture the subjectivities surrounding aging and health promotion for individuals in this age group [18,22].

Regarding the sample characteristics, there was a predominance of women (*n* = 11) with a mean age of 68 years, most of whom were retired and living with family members. This profile aligns with data from the Brazilian Institute of Geography and Statistics [2], which highlights both the ongoing population aging and the feminization of old age in Brazil [2]. These findings also corroborate Cepellos [25], who observed that women tend to engage more in health promotion activities than men, as they often seek strategies for self-care and family care within their social context.

In the first theme identified through thematic analysis, “Health, aging, and quality of life,” the significant presence of chronic conditions such as hypertension, diabetes, and musculoskeletal disorders was noted, confirming a pattern of morbidity reported in studies on older populations [26,27]. It is important to emphasize that the aging process promotes numerous changes in individuals, including physiological declines in the nervous and musculoskeletal systems [28], which subsequently affect functional capacity and contribute to the development of chronic diseases [29].

Nevertheless, despite these conditions, participants reported a positive perception of their health, viewing older age as a distinct stage of life in which, through certain actions, they can achieve a good quality of life and well-being. In this regard, health was not solely associated with the absence of disease but was also linked to well-being and other subjective contexts. Canguilhem [30] discusses this perspective, showing that health status is deeply interconnected with social and cultural aspects, constituting a socially and subjectively constructed process, which cannot be defined solely by biological markers or a biomedical lens. This understanding aligns with the expanded concept of health, which views the health–disease process as the result of the interaction between multiple dimensions, including biological, psychological, economic, cultural, social, individual, and collective factors, placing the individual at the center of these dimensions [31].

Family relationships, in turn, emerged as a significant factor in well-being, cited as a source of emotional, functional, and social support, as well as motivation to seek health promotion strategies, such as practicing Tai Chi in this study. These perceptions reinforce the findings of studies, such as those by Veras and Oliveira [32], who argue that engagement in meaningful activities strengthens self-esteem and social belonging among older adults. Furthermore, emotional and instrumental support provided by family and friends can be decisive for adherence to health promotion programs [3,33].

Autonomy in daily activities, a sense of well-being, family interaction, and physical/mental vitality were highly valued elements when participants described their quality of life. These findings are consistent with the active aging framework proposed by the World Health Organization [34], which emphasizes the importance of social participation, safety, and health for quality aging. It is noteworthy that, from the participants’ perspective, the concepts of quality of life and health are often treated as interconnected and synonymous. This overlap has also been observed in other studies, especially considering that, although both concepts share “concrete” elements that influence their perception [35], such as biological components related to health, they are also influenced by subjective variables, including self-perception during the aging process. We also highlight that in the participants’ discourse, health and quality of life appear as overlapping concepts, since both are understood as conditions that allow one to “live well,” with autonomy and well-being. Both the perception of health and that of quality of life are subjective, linked to self-assessment, and extend beyond biological aspects; an interrelation with emotional aspects is also perceived.

The second theme, “Involvement and engagement with physical activity,” obtained from the thematic analysis, revealed that all participants regularly engaged in physical activity and held a positive understanding of its benefits. It is worth noting that our sample consisted of participants enrolled in a university-based guided physical activity program, which included Tai Chi and supervised walking sessions led by physical education professionals.

Beyond the physical gains derived from this involvement, such as the control of chronic diseases and improvements in strength and balance, participants reported emotional and social benefits, including reduced anxiety and stress, as well as an increased sense of belonging. Regarding this aspect, regular physical activity is essential for maintaining both physical and mental health in later life [36,37]. The literature further supports its relevance for healthy aging, particularly in the roles of fall prevention, cognitive improvement, and reduction in depressive symptoms [38,39]. Considering walking and Tai Chi specifically, studies have shown the benefits of these practices on various aspects of practitioners’ health, particularly mental health [40]. The possibility of social interaction, interaction with the environment, and understanding one’s own physical capabilities emerge as key elements to consider, regardless of age group.

The main motivators for adherence to the physical activity program ranged from family encouragement and personal interest to social interaction and the pursuit of autonomy as a means of coping with the effects of aging. These findings align with other studies that report personal motivations as key factors for adherence to physical activity programs [15,41].

On the other hand, important barriers to access physical activity programs emerged, including social, economic, biological, and cultural factors. Given that most participants were retired and had limited income, economic barriers were the most frequently reported. This group depends on free public initiatives to sustain their participation, which reflects broader inequities in access to health promotion services. Physical limitations (e.g., pain, injuries, comorbidities), lack of time due to family responsibilities, and service disruptions (e.g., during the COVID-19 pandemic) were also cited as obstacles, consistent with findings from other studies [6,33,42,43]. Spiteri and collaborators [41] identified similar barriers in a qualitative review, including concerns about physical health/fitness, lack of motivation or interest, fear or history of falls, and environmental challenges. Key motivators included family/friend support, social interaction, and perceived personal benefits.

These findings align with WHO reports, which emphasize the need to expand equitable access to health promotion services in both urban and rural settings for diverse populations [44], particularly among older adults [45]. Such barriers underscore the importance of inclusive, intersectoral public policies that address the specific needs of this population [45,46].

The third theme, “Perspectives on health promotion strategies,” highlighted the high value placed on participation in social programs, especially those offered by universities and municipal governments, based on the sample considerations. Most participants reported improvements in their health, autonomy, and physical capacities, as well as positive perceptions of quality of life and health, and expanded support networks and social interactions. In addition to the inherent benefits of physical activity, interaction with professionals and intergenerational engagement were perceived as capable of fostering positive changes in well-being and self-concept, consistent with other studies [15,47,48].

Regarding healthcare access, participants reported relying primarily on the Brazilian SUS, with both positive and negative experiences. SUS is the country’s largest public health policy, based on universality, comprehensiveness, and equity, aiming to ensure access to health for all citizens [49]. It operates through three levels of care: (a) primary (basic health units and clinics focused on prevention and health promotion), (b) secondary (specialized outpatient services), and (c) tertiary (specialized hospital-based services). Most participants used primary care for managing and monitoring their conditions, given their lower complexity. In some cases, they need secondary care, where they reported delays in service delivery, bureaucratic hurdles in scheduling, and insufficient information about access [49].

These findings reveal gaps in the comprehensiveness of SUS and reinforce the need for greater investment in secondary and tertiary care, with an emphasis on healthy aging [45]. The perception of bureaucracy and lack of preparedness in service provision, as reported by some participants, also signals the need for more humanized care and adequate professional training to address the specific demands of older adults [50]. The National Health Policy for Older Persons seeks to address these gaps, but its effectiveness still depends on professional training, service organization, and intersectoral collaboration [32,50].

The lack of effective dissemination of information compromises access to initiatives for a large portion of the population that could significantly improve their quality of life. Social communication strategies and engagement with community leaders could be effective mechanisms to broaden awareness of these opportunities [51].

When older adults are included in supportive contexts, such as well-structured physical activity programs and social interaction spaces, they become active agents in their own health, demonstrating a willingness to participate, engage in lifelong learning, and collectively construct their well-being [46]. This underscores the need for integrated public policies focused on promoting autonomy, social inclusion, and valuing old age as a productive stage of life. Strategies for “active aging,” as advocated by WHO, depend not only on individual behavior but also on institutional commitment to equity, accessibility, and social justice [35].

It is important to highlight in this section that there is a distinction between engagement with physical activity and perspectives on health promotion strategies, as presented in the results section. Engagement involves an analysis centered on individual behavior and adherence conditions, demonstrating how older adults experience health promotion in practice as a means of active aging through exercise and socialization [34]. Perspectives, on the other hand, are more comprehensive and reflective, exploring how older adults perceive the set of health promotion actions and policies, not only related to exercise, but also the programs, institutions, and their support networks [15].

It is essential to note that the results presented can also be interpreted from the perspective of Dahlgren and Whitehead’s model of social determinants of health [52], which encompasses factors ranging from individual-level to broader socioeconomic and political conditions. Biological factors related to the subjects, such as the predominantly female sample, with an average age over 65 years, and the presence of chronic degenerative diseases, are not able to interfere with the perception of health, showing that it is socially constructed and more directly related to other lifestyle factors, such as the practice of physical activity, for example.

The adoption of a more active lifestyle is an individual choice and is intimately linked to cultural, social, and economic factors, representing opportunities or barriers for each individual, depending on the context in which they are situated. Considering the context of social and community support, family and other social groups have emerged as important tools for promoting adherence to a more active lifestyle and enhancing well-being. However, it was possible to identify that living conditions and available resources directly influence this group’s access to health promotion strategies, as they are retired older people with more limited incomes, making it necessary for them to benefit from intersectoral policies that promote equity [5,9].

Furthermore, the results also reflect the Brazilian context in which population aging and inequalities can shape access to health promotion strategies, considering that health is a collective and political product, determined by social and cultural factors, and the creation of environments favorable to active aging will directly reverberate in the well-being of the older population.

## 5. Conclusions

This qualitative study revealed that older adults participating in a health promotion initiative centered on physical activity held a positive perception of their health and quality of life, even in the presence of comorbidities. Regular participation in physical activities, particularly in structured, supervised, and socially integrated contexts, emerged as a key factor in promoting physical, emotional, and social well-being, despite the existence of multiple barriers to access, most notably of a socioeconomic nature.

The findings emphasize the importance of understanding aging beyond biomedical aspects, considering lived experiences, subjectivities, and the social contexts that influence older adults’ daily lives. The discussion presented here highlights the urgency of viewing aging as an active and participatory process, with investments in strategies that foster health, social inclusion, and equitable access to public policies.

Nonetheless, substantial challenges persist in accessing health promotion services and programs, which remain marked by social inequities and structural limitations, such as insufficient information and bureaucratic barriers within the SUS. There is an urgent need for integrated and intersectoral public policies that are sensitive to the specific needs of older adults, ensuring the continuity and expansion of initiatives such as those portrayed in this study, and recognizing older persons as rights-bearing individuals capable of actively contributing to the construction of their own health and citizenship.

Studies like this show, in the voices of older adults themselves, that the perception of health and quality of life depends on multiple factors (social, cultural, political, economic, and biological). Furthermore, the success of public policies aimed at aging depends on promoting equity in access to health promotion programs, creating community spaces for social interaction and physical activities, expanding funding and reach of community programs, improving the dissemination of public initiatives, and providing professional training that is more sensitive to social realities.

This study, employing a qualitative approach, involved a limited number of older individuals from the same context, with a predominantly female sample. This fact may restrict the generalization of the results to other older populations with different socioeconomic, cultural, or geographic profiles. For this reason, inferences about cause-and-effect relationships are not possible. Furthermore, only the older individuals who continued in the physical activity program after the clinical trial were included, and the results may reflect more positive perceptions, since more engaged and motivated individuals tend to remain in such programs. Finally, to increase understanding of the phenomenon, it is necessary to utilize other data sources beyond focus groups, thereby enhancing data triangulation through various methods.

## Figures and Tables

**Table 1:** Questions included in the focus group script.

1. Could you tell us your age, who you live with, and what your profession is/was?
2. What are your main motivations for engaging in physical activity? And what are the main barriers you face?
3. What kinds of physical activities are part of your daily routine?
4. What role does physical activity play in your life?
5. How do you evaluate your health and quality of life?
6. How do you usually use health services, and how do you evaluate them?
7. What are the main benefits of participating in a supervised physical activity program?

**Table 2:** Sociodemographic characteristics of the participants of the Focus Groups.

ID	Sex	Age	Marital Status	Occupation	Focus Group
ID1	Male	70 years	Single	Retired	G1
ID2	Male	71 years	Married	Retired	G1
ID3	Female	68 years	Married	Retired/Hairdresser	G1
ID4	Female	65 years	Widowed	Retired/Domestic worker	G1
ID5	Female	70 years	Widowed	Pensioner	G1
ID6	Female	73 years	Widowed	Retired	G1
ID7	Female	65 years	Single	Retired	G1
ID8	Female	65 years	Divorced	Retired	G2
ID9	Female	69 years	Divorced	Retired	G2
ID10	Male	76 years	Widowed	Retired	G2
ID11	Female	63 years	Divorced	Retired	G2
ID12	Female	68 years	Single	Retired	G2
ID13	Female	70 years	Single	Retired	G2
ID14	Female	62 years	Married	Salaried worker	G2

Note: G1 and G2 refer to Focus Group 1 and Focus Group 2, respectively.

**Table 3:** Themes and subthemes identified through thematic analysis.

Themes	Subthemes
1. Health, aging, and quality of life	1.1 Presence of illnesses and comorbidities 1.2 Perspectives on the aging process, quality of life, and health
2. Engagement with physical activity	2.1 Understanding of its importance and benefits 2.2 Motivation for practice 2.3 Perceived barriers
3. Perspectives on health promotion strategies	3.1 Participation in social programs and projects 3.2 Use of the healthcare system

## Data Availability

Due to privacy and ethical restrictions, the data (audio recordings and transcripts) from this study are not publicly available. De-identified transcripts may be obtained from the corresponding author upon reasonable request.

## Abbreviations

The following abbreviations are used in this manuscript:

SUS	Unified Health System
WHO	World Health Organization
IBGE	Institute of Geography and Statistics
